# Improved Effectiveness of Combined Screening for Multiple Cancers: A Government‐Organized Population‐Based Study in China

**DOI:** 10.1002/cam4.70463

**Published:** 2024-11-28

**Authors:** Qian Lu, Di Liang, Jin Shi, Siqi Wu, Xinyu Du, Yutong He

**Affiliations:** ^1^ Cancer Institute in Hebei Province The Fourth Hospital of Hebei Medical University Shijiazhuang China; ^2^ School of Public Health Hebei Medical University Shijiazhuang China

**Keywords:** cancer, compliance, effectiveness, risk assessment, screening

## Abstract

**Objective:**

The purpose of this study was to analyze the effectiveness of the Cancer Screening Program in Urban China (CanSPUC) in Hebei Province, 2016–2023.

**Methods:**

A questionnaire for risk factors of lung, breast, upper gastrointestinal, liver, and colorectal cancers was administered to urban residents aged 40–74 years in five cities to assess their cancer risk. High‐risk participants were invited for screening and classified into five groups on the basis of the number of cancer types that were assessed to be high risk. The participation and positive outcome rates were analyzed. The incidence and the mortality of five types of cancer and all‐cause mortality of the screened and nonscreened participants were calculated via inverse probability weighting.

**Results:**

A total of 237,975 eligible participants were enrolled in our study and 118,339 participants (49.94%) were assessed to be at high risk for one or more of the five cancer types. The number of screenings performed was 103,824, with a screening participation rate of 40.49%. Among the 57,315 screening participants, 9077 (15.84%) had positive cancer diagnoses and 871 (1.52%) were diagnosed with suspected cancer. Compared with the participants at high risk for a single cancer type, the participation and positive outcome rate increased by 45% and 71.5% in the participants at high risk for multiple cancer types. Compared with the non‐screened participants, the screened participants had a 27.0% decrease in mortality due to the five types of cancer and a 45.8% decrease in all‐cause mortality.

**Conclusion:**

A combined screening program for multiple cancers could increase participation and positive outcome rates. It could also decrease the five types of cancer mortality and all‐cause mortality. Our findings highlight the effectiveness of combined screening for multiple cancers with limited health care resources, and may provide foundational evidence for the feasibility of conducting combined screening programs.

## Graphical abstract

We evaluated the effectiveness of the combined screening mode in Hebei Province. We found that individuals at high‐risk for multiple cancer types had higher participation and positive rates than those at high‐risk for a specific single‐cancer type. Screened participants had a lower mortality rate than non‐screened participants.

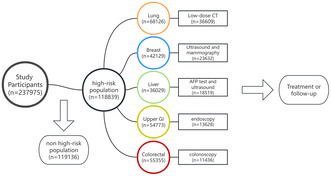

## 1 Introduction

GLOBOCAN 2022 reported that there were close to 20 million new cases of cancer in 2022 as well as 9.7 million cancer deaths [1]. There were approximately 4.8 million new cancer cases and 2.6 million cancer deaths in China. Lung cancer, liver cancer, gastric cancer, colorectal cancer, and esophageal cancer are the top five cancers leading to death and account for 67.5% of all cancer deaths [2]. Sufficient and credible evidence in recent decades has demonstrated that screening reduces the mortality of several common cancers in the USA and other high‐income countries [3]. The US National Lung Screening Trial (NLST) and Dutch–Belgian Lung Cancer Screening Trial (NELSON), reported that low‐dose computed tomography (LDCT) screening for lung cancer was associated with 20% and 24% reductions in lung cancer mortality, respectively [4, 5]. Endoscopic screening has resulted in 23% and 57% decreases in upper gastrointestinal cancer incidence and mortality, respectively [6]. The use of colonoscopies to screen for colorectal cancer reduces the risk of incidence and mortality by 18% and 10%, respectively [7]. The use of mammography and ultrasound screening for breast cancer could reduce the risk of cancer. In a meta‐analysis of 11 randomized trials, the relative risk of breast cancer mortality for women invited for screening compared with controls resulted in a relative risk reduction of 20% [8]. Hepatocellular carcinoma (HCC) via regular liver ultrasound (alone or in combination with serum markers) is beneficial and cost‐effective. Screening can identify more patients with early‐stage HCC and increase their survival time [9, 10].

Due to common cancers having similar risk factors and hereditary susceptibility, combined screening can improve the utilization of health resources and the effectiveness of population management. To date, the Prostate, Lung, Colorectal, and Ovarian (PLCO) cancer screening trial has assessed testing for four cancers simultaneously and can be viewed as a multiphasic cancer intervention [11, 12, 13, 14]. Population‐based research to optimize the screening process (PROSPR) involves conducting multilevel observational research to evaluate factors that affect the quality and outcomes of screening processes for cervical, colorectal, and lung cancer [15]. There are four national cancer screening programs (NCSPs) funded by the Chinese government, which include cervical, breast, colorectal, lung, esophageal, stomach, liver, and nasopharyngeal cancer screenings [3]. A combined screening program for multiple types of cancer is performed in practice, but the cancer screening evaluation is usually performed for a single cancer type. Research on how to evaluate the effectiveness of combined screening comprehensively is limited.

Since 2012, the Cancer Screening Program in Urban China (CanSPUC) has conducted combined screening for lung, colorectal, breast, upper gastrointestinal, and liver cancers. Hebei Province was one of the first provinces to conduct the program. The combined screening program based on a high‐risk questionnaire and the simultaneous screening for five types of cancer achieved notable effects. Our study evaluated the effectiveness of screening, including the participation rate, positive outcome rate, and mortality to provide a foundation for combined screening strategies.

## 2 Materials and Methods

### 2.1 Study Design and Participants

We performed this population‐based study under the framework of the CanSPUC government‐supported cancer screening program initiated in October 2012 in China. Residents aged 40–74 years living in Hebei Province who did not have a history of cancer, kidney dysfunction, or severe disease of the heart, brain or lungs were recruited through a variety of methods, such as telephone calls, personal encounters, social media, and community advertisements. The participants answered a comprehensive questionnaire about their exposure to risk factors and evaluated their cancer risk via a scoring system established by the National Cancer Center and based on the Harvard Cancer Risk Index [16]. High risk to these five types of cancer, including lung, breast, liver, colorectal, and upper gastrointestinal cancers, can be assessed using this questionnaire.

**FIGURE 1:**
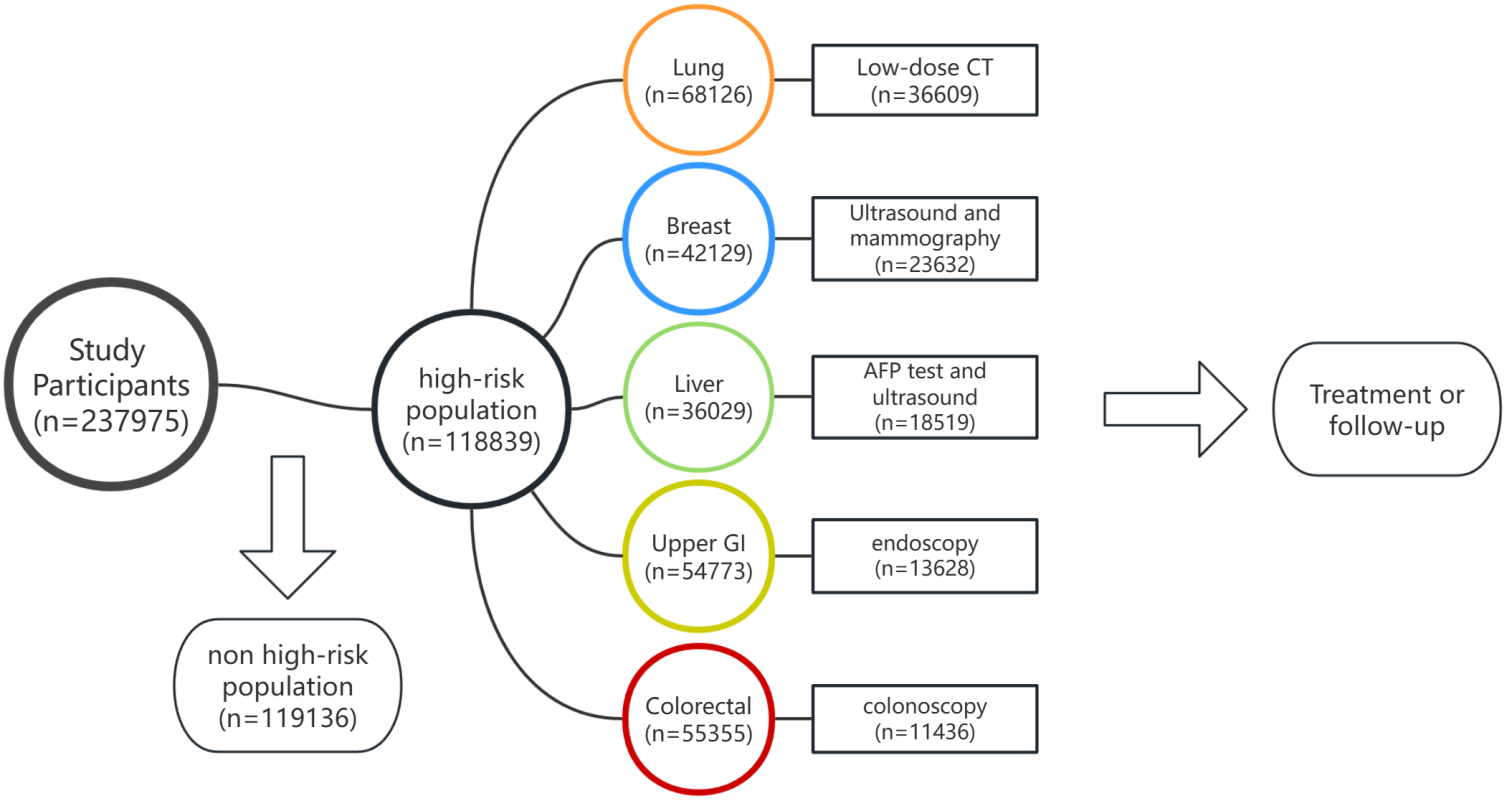
Participant assessment and screening participation results of multi‐cancer combined screening flowchart from five cities in Hebei Province, 2016–2023.

Participants at high risk for lung cancer were recommended to undergo LDCT. Women at high risk for breast cancer were recommended to undergo ultrasound and mammography screening. Individuals at high risk for liver cancer were recommended to undergo alpha‐fetoprotein (AFP) tests and abdominal ultrasound screening. Participants at high risk for colorectal and upper gastrointestinal cancer were advised to undergo colonoscopy and upper gastrointestinal endoscopic screening. Furthermore, abnormalities detected during screening were carefully checked in accordance with standard clinical procedures, and biopsies were taken for pathological diagnosis (Figure 1). This study included participants from 2016 to 2023 and covered a total of five cities (Shijiazhuang, Tangshan, Xingtai, Handan, and Baoding) in Hebei Province [17]. All participants provided written informed consent. Ethical approval was obtained for all data collection.

### 2.2 Definitions

#### 2.2.1 High‐Risk Group Definitions

The high‐risk population evaluation system has been in use by the National Cancer Center for more than a decade to collect common cancer epidemiology data in China. Through a multidisciplinary expert discussion consensus method and with the “Harvard cancer risk index” as the theoretical basis, a cancer risk assessment model was established to determine high‐risk groups for cancer. The expert panel scored the degree to which risk factors were associated with cancer according to Chinese characteristics. The cumulative risk score is divided by the average risk score of the general population to obtain the final relative risk of an individual. Individuals with a higher relative risk for each cancer type are placed in a high‐risk group [18]. The information used to define the high‐risk groups included demographic characteristics (age, sex, education level, body mass index [BMI], etc.), lifestyle factors (smoking, drinking, physical activity, etc.), family history of cancer, and history of disease (chronic respiratory disease, digestive system disease, hepatobiliary disease, hypertension, diabetes, and hyperlipidemia). The identification of high‐risk populations is the national standard [3], and notably, the assessment criteria for the high‐risk groups for each cancer type are consistent across many years of screening practice. We defined participants at high risk for any specific type of cancer as Group 1. Group 2 was defined as those at high risk of any two types of cancers with 10 different combinations. Group 3 was defined as those at high risk of any three types of cancers with 10 different combinations. Group 4 was defined as those at high risk of any four types of cancers with five different combinations. Group 5 was defined as those at high risk for all five cancer types. All participants who engaged in the risk assessment and were examined were in the screened group, with the remaining being placed in the nonscreened group.

#### 2.2.2 Positive Outcome Definitions

##### 2.2.2.1 Lung Cancer

Nodule images were observed via multirow (64 rows) spiral CT (at least 16 rows). The long and short diameters were measured at the largest section of the nodule with an electronic measuring ruler. According to CanSPUC protocol, the positive nodules detected by LDCT scans were classified as the following: (1) solid or partially solid nodules larger than 5 mm in mean diameter, (2) nonsolid nodules larger than 8 mm in diameter, (3) detected luminal nodules in the trachea, or (4) airway pathology (requiring bronchial biopsy). In addition to positive nodules, lung cancer or suspected lung cancer diagnoses were defined as positive outcomes in our study [19].

##### 2.2.2.2 Breast Cancer

Women at high risk of developing breast cancer were screened via ultrasound and mammography methods. Breast imaging reporting and data system (BI‐RADS) results of Levels 4 and 5 were defined as positive outcomes [20].

##### 2.2.2.3 Liver Cancer

Only participants who were identified as high risk were subjected to alpha‐fetoprotein (AFP) measurement and ultrasonography examinations by uniformly trained medical staff at local qualified clinical centers. Our study defined liver cancer or suspected liver cancer, probed liver‐occupying lesions and cirrhosis as positive outcomes.

##### 2.2.2.4 Upper Gastrointestinal Cancer

Upper gastrointestinal cancer screening included esophageal cancer and gastric cancer screening. Abnormal findings during endoscopy were carefully examined in accordance with standard clinical procedures, and biopsy samples were collected for further pathological diagnosis according to clinical guidelines. In this study, esophageal precancerous lesions referred to squamous epithelial intraepithelial neoplasia or glandular intraepithelial neoplasia that occurred in the esophagus; gastric precancerous lesions referred to glandular intraepithelial neoplasia that occurred in the stomach; esophageal cancer referred to esophageal squamous cell carcinoma adenocarcinoma, adenocarcinoma or other malignant tumors; and gastric cancer referred to adenocarcinoma or other malignant tumors that occurred in the stomach. All of the above factors were considered positive outcomes [21].

##### 2.2.2.5 Colorectal Cancer

Colonoscopy was recommended for those at high risk of colorectal cancer. The final clinical diagnosis was classified according to the latest clinical guidelines for colonoscopy and/or histopathological reports. Abnormal colonoscopy findings were classified according to the fifth edition of the World Health Organization classification of digestive system tumors. Colorectal cancer (CRC) was defined as malignant cancer tissue infiltrating the submucosa of the colon or rectum. Colorectal precancerous lesions were defined as adenomas with at least one of the following characteristics: (1) high‐grade intraepithelial neoplasia of the glandular epithelium; (2) histopathological features of villous or tubular‐villous adenoma; (3) sessile serrated adenoma; or (4) traditional serrated adenoma, tubular adenoma, or serrated polyps that failed to be classified. Both CRC and colorectal precancerous lesions were considered positive outcomes [22].

#### 2.2.3 Outcomes

Primary outcomes focused on the participation and positive outcome rates. The main secondary outcomes were the incidence of the five types of cancer and all‐cause mortality. The main indicators were calculated as follows.

##### 2.2.3.1 High‐Risk Rate

Number of individuals assessed as high risk by questionnaire/Number of individuals completing the questionnaire × 100%.

##### 2.2.3.2 Participation Rate

Number of screenings utilized in high‐risk individuals in a specific time frame/Screening opportunities for high‐risk individuals in the same specific time frame × 100%.

##### 2.2.3.3 Screening Opportunity

Cumulative count of the number of cancer types assessed as high risk for each individual.

##### 2.2.3.4 Positive Rate

Number of individuals with positive outcomes in a specific time frame/Number of individuals screened in the same specific time frame × 100%.

### 2.3 Participant Follow‐Up

All participants were followed up by active and passive methods until December 31, 2023. The status of the participants was retrieved from linkages, including the cancer registry system and death surveillance system, every 6 months. Data from these two provincial linkages have been extensively used to assess disease burden, as well as for other research purposes. For the screened group, survival status was regularly monitored according to clinician recommendations. To control potential immortal time bias, the date of entry for the nonscreened participants was the date of the questionnaire assessment, and the date of entry for the screened participants was the date of screening. Starting from the cohort entry date, the follow‐up time for the occurrence of the five types of cancer was measured until the earliest time of the incidence, death, or censoring of the five types of cancer. The time from cohort entry to the event of death, whether due to the five types of cancer or all‐cause mortality, was determined using either the occurrence of death or the censoring date, depending on which occurred first. Newly diagnosed cases of cancer were classified by site according to the International Statistical Classification of Diseases, Tenth Revision. All positive patients received standardized and rationalized treatment.

### 2.4 Quality Control and Statistical Analysis

A quality control team was established to review all cancers and suspected cancers, and 1% of negative results were sampled for review. The categorical variables, including the baseline demographics and disease characteristics, are presented as frequencies and proportions. The distribution between groups was assessed via the chi‐square test. The chi‐square test trend was used to test the rate trends. There were no missing data for basic variables (including age, sex, and region). We applied an inverse probability weighting (IPW) approach to calculate standardized differences in the screened and non‐screened participants. First, we performed a multivariate logistic regression analysis (dependent variable, participation in any screening; independent variables, baseline characteristics, including age, sex, and region) to predict the likelihood of participants undergoing screening. Second, we calculated the weights of each individual of the inverse of the probability for screened participants (those who received a screening) and non‐screened participants (those who did not receive a screening). This method was used to balance the baseline characteristics to evaluate the effectiveness of screening among participants. All the statistical analyzes were performed using the R version 4.2.1 software package. A two‐tailed *p* < 0.05 was considered statistically significant.

## 3 Results

### 3.1 High‐Risk Evaluation and High‐Risk Rate

A total of 237,975 participants in Hebei Province were assessed via questionnaires from 2016 to 2023. A total of 118,839 individuals were assessed as high risk for cancer (a specific type of cancer or multiple types of cancer), with an overall high‐risk rate of 49.94%. A total of 256,412 times of high‐risk were evaluated for all cancer types. The high‐risk rates for lung, breast, liver, upper gastrointestinal, and colorectal cancers were 28.63%, 17.70%, 15.14%, 23.02%, and 23.26%, respectively. The high‐risk rate was higher in women (50.41%) than in men (49.31%) (*p* < 0.001). Additionally, the high‐risk rates significantly increased with increasing age (*p* trend < 0.001) (Table 1). The high‐risk rates were highest in the 65–69 years age group for men and 55–59 years age group for women. The high‐risk rates were lowest in the 45–49 years age group for both sexes (Figure S1a).

**TABLE 1:** Characteristics and participation rate of study population in different groups.

Variable	Study population	High‐risk population	High‐risk rate (%)	*χ* ^2^	*p*	Number of screenings	Participation rate (%)	*χ* ^2^	*p*
	*N* (%)	*N* (%)				*N* (%)			
Gender									
Male	103,044 (43.3)	50,815 (42.8)	49.31	28.28	< 0.001	35,461 (34.2)	34.72	2349.36	< 0.001
Female	134,931 (56.7)	68,024 (57.2)	50.41			68,363 (65.8)	44.31		
Age group (years old)									
40–44	20,271 (8.5)	6334 (5.3)	31.25	1762.17	< 0.001	6790 (6.5)	45.80	2717.97	< 0.001
45–49	35,272 (14.8)	15,555 (13.1)	44.10			14,567 (14.0)	44.66		
50–54	40,159 (16.9)	20,836 (17.5)	51.88			21,345 (20.6)	45.10		
55–59	39,839 (16.7)	22,210 (18.7)	55.75			21,405 (20.6)	43.03		
60–64	41,684 (17.5)	21,887 (18.4)	52.51			19,703 (19.0)	41.64		
65–69	38,892 (16.3)	21,975 (18.5)	56.50			14,698 (14.2)	32.55		
70–74	21,858 (9.2)	10,042 (8.5)	45.94			5316 (5.1)	27.37		
High‐risk group									
Group 1*	237,975 (100.0)	48,909 (41.2)	20.55	46,840.28	< 0.001	14,910 (14.4)	30.49	1617.58	< 0.001
Group 2*		30,430 (25.6)	12.78			25,511 (24.6)	41.92		
Group 3*		19,144 (16.1)	8.04			24,301 (23.4)	42.31		
Group 4*		12,569 (10.6)	5.28			21,862 (21.1)	43.48		
Group 5*		7787 (6.6)	3.27			17,240 (16.6)	44.28		
Total		118,839 (100.0)	49.94			103,824 (100.0)	40.49		

* Group 1 was defined as those at high risk of any specific type of cancer. Group 2 was defined as those at high risk of any two types of cancers. Group 3 was defined as those at high risk of any three types of cancers. Group 4 was defined as those at high risk of any four types of cancers. Group 5 was defined as those at high risk for all five cancer types.

In all high‐risk participants, 48,909 high‐risk participants (41.2%) were evaluated to be high risk for one specific type of cancer; 30,430 high‐risk participants (25.6%) were evaluated to be high risk for any two types of cancer; and 19,144 (16.1%), 12,569 (10.6%), and 7787 (6.6%) high‐risk participants were evaluated to be at high risk for any three, four, or five types of cancer, respectively. Among all the study participants, 20.55% had a high risk for one type of cancer, and 29.39% had a high risk for two or more types of cancer. Among the 10 different combinations associated with a high risk of any two types of cancer, the lung/breast combination was the most common, with 6088 participants, followed by the lung/colorectal combination, with 5142 participants. A total of 5077 participants were evaluated to be at high risk for lung/upper GI/colorectal cancer, which was the most common type among the 10 different combinations of high risk factors for any three types of cancer. The lung/liver/upper GI/colorectal combination was the most common combination among the 5 different combinations associated with a high risk of any four types of cancer, with 7429 participants. Notably, compared to high‐risk female participants, the number of high‐risk male participants was higher only in Group 4 (Figure S1b). Each participant was offered the same number of screening opportunities as the number of cancer types assessed as high risk (Table S1).

### 3.2 Participation Rates

A total of 103,824 screenings were performed on 57,315 screened individuals. For the number of individuals assessed as high risk for all cancer types (screening opportunities), the participation rate was 40.49%. There were 21,211 men (37.0%) and 36,104 women (63.0%) who participated in screening (a specific type of cancer or multiple types of cancer), and women had a higher participation rate (44.31% vs. 34.72%; *p* < 0.001). A total of 53.74%, 56.09%, 51.40%, 24.88%, and 20.66% of participants at high risk for lung cancer, breast cancer, liver cancer, upper gastrointestinal tract cancer, and colorectal cancer, respectively, participated in specific cancer screenings. Trends in participation rates decreased with age group and increased with the number of high‐risk cancer types (*p* trend < 0.001). The participation rate was highest in Group 5, with a value of 44.28%. Compared with those of Group 1, the participation rates of Groups 2–5 were 11.43%, 11.82%, 12.99%, and 13.79% higher in absolute value, respectively, exhibiting participation rate increases of 37.49%, 38.77%, 42.60%, and 45.23%, respectively (Table 1).

**FIGURE 2:**
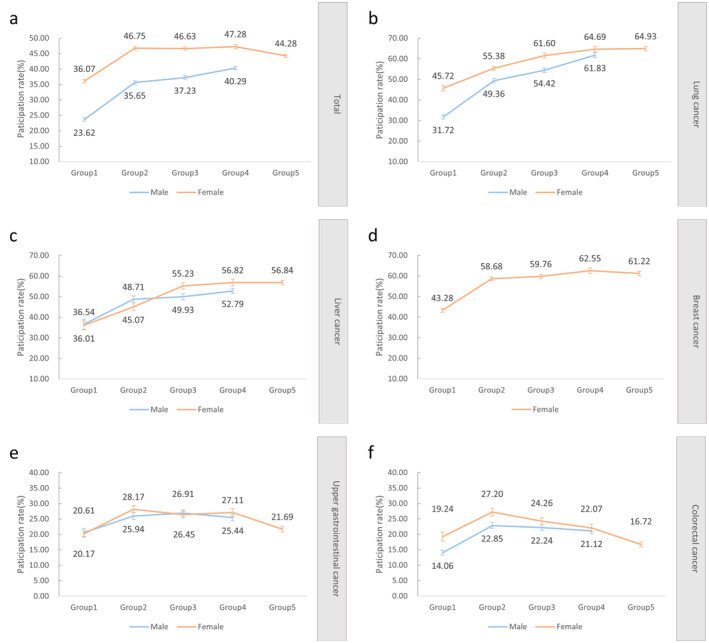
Overall participation rates and participation rates for each cancer type among men and women in different high‐risk groups. (a) Overall participation rate. (b) Lung cancer participation rate. (c) Liver cancer participation rate. (d) Breast cancer participation rate. (e) Upper gastrointestinal cancer participation rate. (f) Colorectal cancer participation rate. * Group 1 was defined as those at high risk of any specific type of cancer. Group 2 was defined as those at high risk of any two types of cancers. Group 3 was defined as those at high risk of any three types of cancers. Group 4 was defined as those at high risk of any four types of cancers. Group 5 was defined as those at high risk for all five cancer types.

The participation rates of those in the groups with multiple types of cancer (Groups 2–5) were higher than those in Group 1 for both sexes. The participation rates of women were higher than those of men in each high‐risk group (Figure 2a). When each cancer type was analyzed separately, the lung cancer participation rate increased with the number of high‐risk cancer types in both sexes (Figure 2b). The participation rate of those with high risk for liver cancer also increased and was highest for women in Group 5 (56.84%) and for men in Group 4 (52.79%) (Figure 2c). The participation rate of those with high risk for breast cancer was highest in Group 4 (62.55%) (Figure 2d). The participation rate of those with high risk for upper gastrointestinal cancer was highest in Group 3 for men (26.91%), whereas it was highest in Group 2 for women (28.17%) (Figure 2e). The participation rate of those with high risk for colorectal cancer were highest in Group 2 for both sexes (Figure 2f). Notably, the participation rates of those with high risk for upper gastrointestinal cancer and colorectal cancer were lower than those with high risk for the other three types of cancer.

**FIGURE 3:**
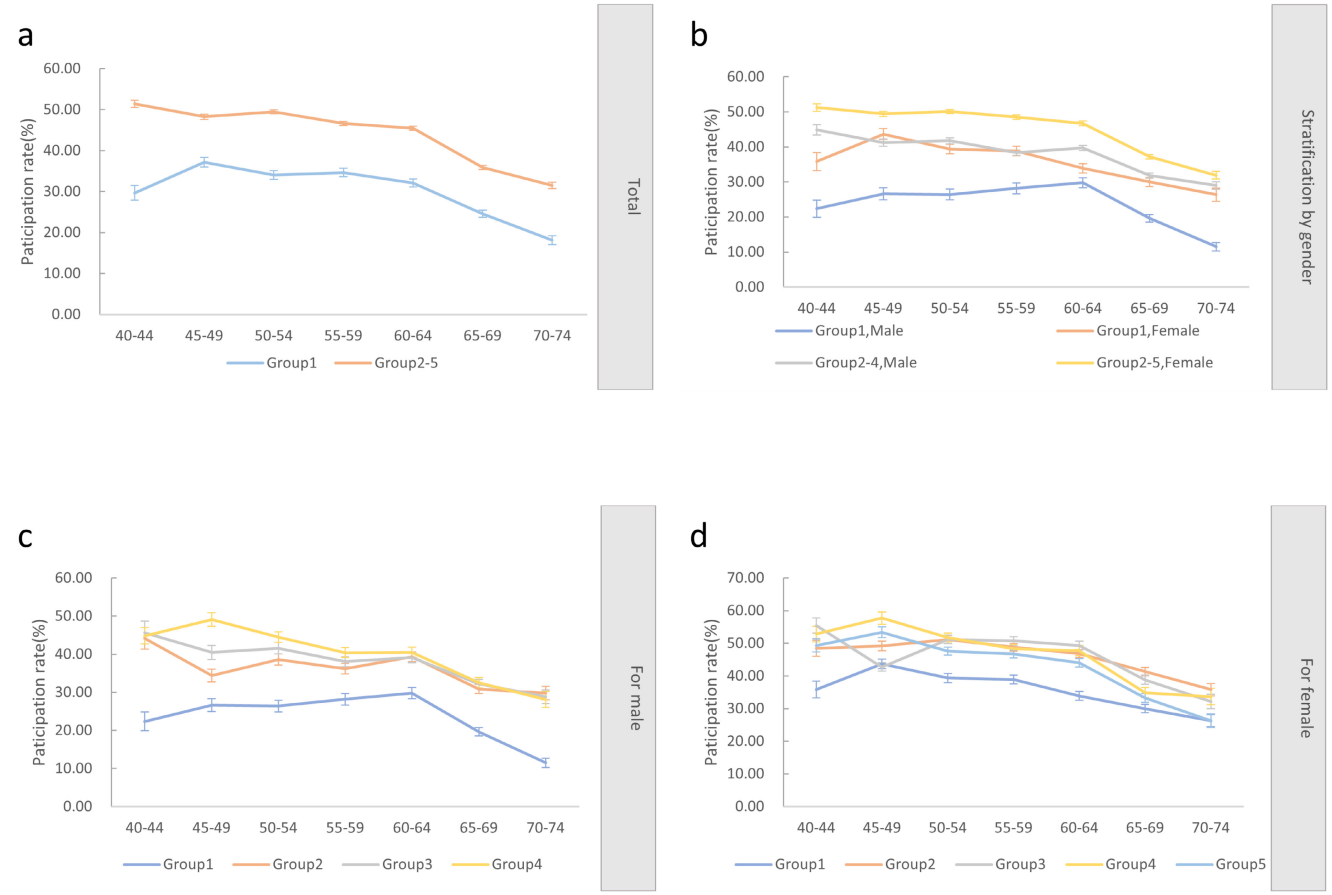
Participation rates for each age group by gender and high‐risk group. (a) Participation rates of Group 1 and Groups 2–5 by age group. (b) Participation rates by age group for men and women in Group 1 and Groups 2–5. (c) Male participation rates by age group in different high‐risk groups. (d) Female participation rates by age group in different high‐risk groups.

In all age groups, participation rates were higher in Groups 2–5 than in Group 1. Older individuals were less likely to participate in screening (Figure 3a), and stratification by sex still yielded this result. Women in Groups 2–5 had the highest participation rates in all age groups, and men in Group 1 had the lowest participation rates in all age groups (Figure 3b). Men in Group 4 had the highest participation rate in the 45–49 years age group (49.11%), and those in Group 1 had the lowest participation rate in the 70–74 years age group (11.50%) (Figure 3c). The female participation rates were the lowest in Group 1, except for those in the 45–49 years and 70–74‐year‐old age groups (Figure 3d).

### 3.3 Positive Outcome Rate

Among the 57,315 screening participants, 9077 (15.84%) were diagnosed with a positive outcome (a specific type of cancer or multiple types of cancer), and 871 (1.52%) were diagnosed with suspected cancer. The positive outcome rates for lung, breast, liver, upper gastrointestinal, and colorectal cancers were 14.88%, 4.12%, 0.94%, 3.22%, and 21.34%, respectively, and the suspected cancer detection rates were 1.32%, 1.17%, 0.03%, 0.36%, and 0.51%, respectively.

**FIGURE 4:**
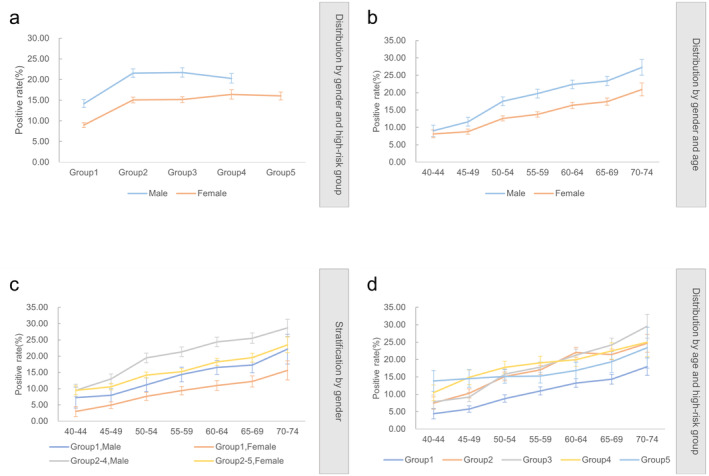
Positive rates in different high‐risk groups and age groups. (a) Overall positive rates for male and female in different high‐risk groups. (b) Positive rates for men and women in different age groups. (c) Positive rates by age group for men and women in Group 1 and Groups 2–5. (d) Positive rate of participants with high‐risk groups in each age group.

The positive outcome rate was higher in men (19.51%) than in women (13.68%) (*p* < 0.001). The positive outcome rate was highest in the 70–74 years age group (23.80%) and lowest in the 40–44 years age group (8.43%). The positive outcome rates significantly increased with increasing age (*p* trend < 0.001). Compared with Group 1, Groups 2–5 had positive outcome rates that were 6.79%, 7.05%, 7.68%, and 5.26% higher in absolute value, respectively, exhibiting positive outcome rate increases of 63.05%, 65.46%, 71.31%, and 48.84%, respectively (Table 2). The positive outcome rates in Group 1 were the lowest for both sexes. The highest positive outcome rate for men was in Group 3 (21.72%), and that for women was in Group 4 (16.38%) (Figure 4a). Men had higher positive outcome rates than women did in all age groups. Older participants were more likely to have positive outcome results (Figure 4b). When stratified by sex, the high‐risk groups for multiple cancer types still had higher positive outcome rates than Group 1 (Figure 4c). The positive outcome rate of Group 1 was the lowest among all age groups (Figure 4d).

**TABLE 2:** Characteristics of positive population in different groups.

Variable	Screening population	Positive population	Positive rate (%)	*χ* ^2^	*p*
	*N* (%)	*N* (%)			
Gender					
Male	21,211 (37.0)	4139 (45.6)	19.51	341.45	< 0.001
Female	36,104 (63.0)	4938 (54.4)	13.68		
Age group (years old)					
40–44	3463 (6.0)	292 (3.2)	8.43	723.07	< 0.001
45–49	8188 (14.3)	791 (8.7)	9.66		
50–54	11,201 (19.5)	1589 (17.5)	14.19		
55–59	11,604 (20.2)	1832 (20.2)	15.79		
60–64	10,920 (19.1)	2057 (22.7)	18.84		
65–69	8670 (15.1)	1738 (19.1)	20.05		
70–74	3269 (5.7)	778 (8.6)	23.80		
High‐risk group					
Group 1	14,910 (26.0)	1606 (17.7)	10.77	173.38	< 0.001
Group 2	16,163 (28.2)	2839 (31.3)	17.56		
Group 3	11,935 (20.8)	2127 (23.4)	17.82		
Group 4	8725 (15.2)	1610 (17.7)	18.45		
Group 5	5582 (9.7)	895 (9.9)	16.03		
Total	57,315 (100.0)	9077 (100.0)	15.84		

### 3.4 Incidence, Mortality and All‐Cause Mortality in the Different Groups

**FIGURE 5:**
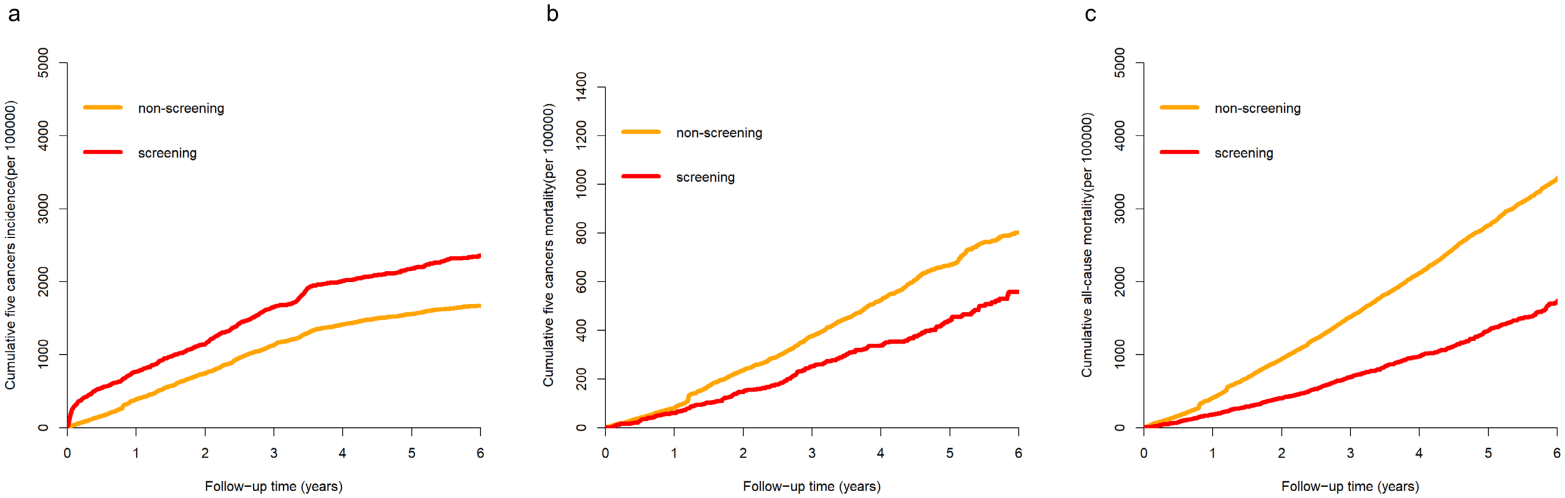
Follow‐up results. (a) Cumulative five types of cancer incidence. (b) Cumulative five types of cancer mortality. (c) Cumulative all‐cause mortality.

Through a median follow‐up of 4.6 years, 1613, 743, 310, 475, and 584 cases of lung, breast, liver, upper gastrointestinal, and colorectal cancer, respectively, were diagnosed. The total follow‐up time was 1,058,650 person‐years. After follow‐up, 1109 (1.93%) patients in the screened group were eventually diagnosed with one of the five types of cancer, 644 (1.12%) resulted in all‐cause mortality, and 210 (0.37%) died from one of the five types of cancer. In the nonscreened group, 2531 (1.40%) patients were diagnosed with one of the five types of cancer, 4549 (2.52%) resulted in all‐cause mortality, and 1084 (0.60%) died from one of the five types of cancer. The cumulative incidence of the five types of cancer was greater in the screened group than in the nonscreened group (Figure 5a). The cumulative mortality of the five types of cancer was lower in the screened group than in the nonscreened group (Figure 5b). The cumulative all‐cause mortality of the screened group was also lower than that of the nonscreened group (Figure 5c). After IPW, the screened group had a significantly greater incidence of the five types of cancer (45.7% increase) (HR 1.46 [95% CI 1.36–1.56]), a lower mortality of the five types of cancer (28.6% decrease) (HR 0.71 [95% CI 0.62–0.83]), and a significantly lower all‐cause mortality (47.3% decrease) (HR 0.53 [95% CI 0.49–0.57]) than those in the nonscreened group.

## 4 Discussion

Some programs for the combined screening of multiple cancers, including PLCO, PROSPR, and NCSP, have been conducted. However, few studies in these programs have focused on the effectiveness of screening for multiple types of cancer. In this study, we evaluated the participation and follow‐up outcomes of a population‐based combined screening of multiple cancers from five cities in Hebei Province. The results of 237,975 participants who underwent screening based on the CanSPUC project framework were reported. We found that those who were evaluated to be at high risk for multiple cancer types were highly motivated to participate in the screening program. The participation rate of the high‐risk population for multiple cancer types was much higher than that for those evaluated to be high risk for a specific type of cancer. Our study also revealed that a combination of multiple cancer screening modalities performed well, exhibiting increases in positive outcome rates. The positive outcome rate of those who were evaluated to be high risk for a specific type of cancer was 10.77%, and the positive outcome rate increased to 18.45% in for the group at high risk for four cancer types; this was a 71.5% increase in the positive outcome rate. With a 4.6‐year follow‐up time, we found that compared with unscreened participants, screened participants had a decrease in mortality from the five types of cancer and in all‐cause mortality. Studies of the CanSPUC project have focused mainly on the effectiveness of cancer screening for one specific type of cancer [18, 23]. To our knowledge, our study is the first to describe the participation and diagnostic yield for the combined screening of multiple types of cancer, providing important evidence toward the effectiveness of these strategies.

Our study was a part of the CanSPUC project, a population‐based cancer screening program in urban regions initiated by the government of China in which eligible participants were invited to participate in a cancer risk assessment. Since different types of cancer have some common risk factors, the combined risk stratification scoring system for five types of cancer could identify individuals at high risk for multiple types of cancer simultaneously. Poor dietary behaviors or lifestyles, such as smoking; alcohol consumption; obesity, lack of exercise; and a low intake of vegetables, fruits, and cereals not only increase the risk of a particular type of cancer [24] but also generally increase the risk of multiple types of cancer. A pancancer genome‐wide association study in Europe suggested that multiple cancers share biological hallmarks and that there are many common genetic etiologies among cancers [25, 26]. Several high‐penetrance mutations have been shown to exhibit pleiotropy across multiple cancers; for example, BRCA2, a gene involved in DNA repair, has been implicated in cancers of the breast, ovary, pancreas, and prostate [27]. Genome‐wide association studies (GWASs) of individual cancer types have also identified loci associated with multiple cancer types, including 5p15 (TERTCLPTM1L) [28], 6p21 (HLA complex), and 8q24 [29, 30]. The advantages of combined screening for multiple cancers include the ability to detect two or more asymptomatic cancers simultaneously, thus improving the effectiveness of screening. In addition, compared with screening for a single specific cancer, combined screening for multiple cancers may avoid duplicating the collection of baseline information and allow for simultaneous management and follow‐up of the screened population.

Participants who were evaluated to be at high risk for any type of cancer were recommended to receive the corresponding cancer screening intervention according to the protocol. Because all the cancer screenings were free of charge, the participants did not need to take cost into consideration. This study demonstrated a 37%–45% increase in the participation rate for the combined screening of multiple cancers compared with that for specific single‐cancer screening. The participation rate increased according to the type of high‐risk cancer that was identified, such as lung cancer, female breast cancer, or liver cancer. That is, when a participant was evaluated to be at high risk for multiple cancers, they were more inclined to participate in screening. Participation in upper gastrointestinal and colorectal cancer screening was relatively low, which may be related to the fear of discomfort from invasive examinations and anxiety [31, 32]. Our study showed that the participation rates of those who were evaluated to be at high risk for two types of cancer, upper gastrointestinal cancer or colorectal cancer, was higher than those of participants evaluated to be at high risk for only one type of cancer (upper gastrointestinal cancer or colorectal cancer). Previous studies have suggested that factors such as individuals' awareness and knowledge about cancer screening; physicians' knowledge, attitudes and recommendations; and logistic and financial considerations are key factors for successful cancer screening uptake [33]. The increased participation rate was more likely due to the high risk of multiple cancers increasing the awareness of the participants. Alternatively, a high risk of multiple cancers increased individuals' concerns about health and promoted screening behavior.

The effectiveness of a population‐based screening program depends not only on the participation rate but also on the detection rate of the screening technique. Our findings suggest that program for the combined screening of multiple cancers not only increased the participation rate but also resulted in a high yield of positive outcome screening results. Compared with CanSPUC in Henan Province, which detected 0.3% breast cancer and 0.19% upper gastrointestinal cancer, there was a high yield of detection in our program [31, 34]. In addition, the detection rate of colorectal neoplasms was higher than that in Zhejiang Province (0.3%) [35]. Positive results for liver and colorectal cancer were comparable to those reported in population‐based screening programs devoted to a single type of cancer [32, 36]. A multidisease screening study in Taiwan (KCIS) found that the combined screening mode improved the effectiveness of screening in terms of participation and positive outcome detection, which was similar to our findings [37]. Furthermore, the simultaneous identification of a large proportion of presymptomatic cases may lead to a greater reduction in mortality than the identification of a single type of cancer. A Keelung community‐based multidisease screening program indicated that 70% attendance to a multiple‐screening program led to a 34% reduction in mortality from cancer, whereas 30% attendance to a single‐screening program led to only a 15% reduction in mortality [38]. The advantages of a multicancer screening program over a specific single‐cancer screening program potentially include a simultaneous identification of two or more asymptomatic cancers, a potential increase in screening participation rates, and a decrease in duplicated effort when performing a community‐based screening activity [37].

The effectiveness of screening strategies must ultimately be demonstrated by incidence and mortality indicators. In our study, after a median follow‐up of 4.6 years, we found that the incidence of the five types of cancer was significantly higher in the screened participants than in the nonscreened participants. Additionally, the mortality of the screened participants was significantly lower than that of the nonscreened participants. This finding is similar to the results of other teams' studies on single‐cancer screening [6, 23]. We also found that screening was more beneficial for all‐cause mortality than for mortality from the five types of cancer. This finding is consistent with a Chinese study on lung cancer screening [39] and may be attributed to the elevated health awareness and adherence rates among study participants. In terms of organization, detecting other disease aspects beyond cancer will also facilitate timely intervention and treatment. Cancers detected by screening had a more favorable stage distribution than cancers that were detected by other methods [40]. Early detection is essential for cancer screening to reduce mortality rates. The proportion of hospitalized Chinese patients with diagnosed Stage II–IV lung, breast, and colorectal cancers is greater than that in the United States, so early detection of cancer in China is urgently needed to reduce mortality rates [41]. The development of a combined screening strategy can simultaneously fulfill the need for the early detection of several cancer types. The follow‐up results of our study may provide a foundation for the development of a combined screening strategy.

Evidence from a population‐based screening study is imperative for comprehensively evaluating the real‐world effectiveness and health economic value of screening for high‐burden cancers in China (esophageal, gastric, lung, colorectal, liver, and breast cancers). This study could highlight practical considerations for exploring screening models. With advancements in precision medicine, the future of cancer screening should shift toward a phase characterized by precision and individualization. In short, targeted “multistage” screening and “multicancer” monitoring are carried out on the basis of more accurate risk assessment tools and stratification criteria, identifying and managing high‐risk individuals that exhibit premalignancy during screening. Such an approach minimizes ineffective or inefficient screening, reduces collateral harm, and ultimately lowers overall costs while improving screening effectiveness and efficacy. It is crucial to have a thorough understanding of various screening modalities (specific single‐cancer screening, the combined screening of multiple cancers, or risk stratification‐based precision screening) and integrate them on the basis of local conditions, population characteristics, and cancer types.

Our study has several limitations. First, we evaluated the effectiveness of the combined screening for five types of cancer in 237,975 participants from five cities in Hebei Province. Although these participants were an important part of the CanSPUC project, their participation may result in selection bias to some extent. The necessity to rapidly and extensively recruit participants for this study constrained the sampling method, potentially resulting in a sample that is not fully representative of the regional population. Furthermore, since the study was based on a national program, the combined screening for multiple cancers included only the five types of cancer specified in the guidelines. Other cancers with a relatively high incidence, such as cervical and prostate cancer, were not included in our screening program. Finally, the combined screening for multiple cancers may also result in false‐positive findings, such as overdiagnosis and unnecessary diagnosis (lead time without benefit). The risk factors associated with screening for multiple cancers have not been explored. In future studies, we will increase the coverage and continue to follow the cohort to monitor long‐term results. Pancancer biomarkers for multicancer risk prediction models should be included to optimize the target population for multicancer screening. Considering the demographic characteristics of the high‐risk cancer population and the imbalance of medical facilities in various regions of China, a multicancer screening strategy has great research value.

## 5 Conclusion

In summary, in this large‐scale program for the combined screening of multiple cancers in China, the effectiveness of screening was greater in the group at high risk for multiple cancers than in the group at high risk for a single cancer. A program for the combined screening of multiple cancers not only increases the participation rate but also results in a high yield of positive outcome screening results. Through follow‐up, the screened participants had significantly lower mortality rates than the nonscreened participants. Further efforts to optimize the screening strategy with more accurate risk assessment tools to identify high‐risk individuals for multiple cancers are highly needed. Our results indicate that combined screening for multiple cancers can maximize the use of limited health care resources and may provide useful information for implementing effective population‐based programs for the combined screening of multiple cancers.

## Author Contributions

**Qian Lu:** data curation (equal), formal analysis (equal), software (equal), visualization (equal), writing – original draft (equal). **Di Liang:** data curation (equal), formal analysis (equal), funding acquisition (equal), writing – original draft (equal), writing – review and editing (equal). **Jin Shi:** formal analysis (equal), software (equal), supervision (equal), writing – review and editing (equal). **Siqi Wu:** data curation (equal), formal analysis (equal), software (equal), writing – review and editing (equal). **Xinyu Du:** conceptualization (equal), data curation (equal), methodology (equal). **Yutong He:** conceptualization (equal), funding acquisition (equal), methodology (equal), project administration (equal), supervision (equal), writing – review and editing (equal).

## Ethics Statement

This study was approved by the ethics committee of the Fourth Hospital of Hebei Medical University. The patients/participants provided their written informed consent to participate in this study.

## Conflicts of Interest

The authors declare no conflicts of interest.

## Supporting information


Supporting Information S1.

## Data Availability

The raw data supporting the conclusions of this article will be made available by the authors, without undue reservation.
