# Supplementary material for: Improved Effectiveness of Combined Screening for Multiple Cancers: A Government‐Organized Population‐Based Study in China
Source: Cancer Med. 2024 Nov 28;13(23):e70463. doi: 10.1002/cam4.70463 (PMC11602753; doi:10.1002/cam4.70463)
Supplement: Supplementary file 1 — Supporting Information S1. [file CAM4-13-e70463-s001.docx]

Supplementary Table S1. High-risk and participation in screening by high-risk combination

| Group | Combination | High-risk population | Screening opportunities | Number of screenings | | | | | Total screenings | Participation rate (%) |
| --- | --- | --- | --- | --- | --- | --- | --- | --- | --- | --- |
|  |  |  |  | Lung | Breast | Liver | Upper GI | Colorectal |  |  |
| Group1 | Lung | 14915 | 14915 | 5624 |  |  |  |  | 5624 | 37.71 |
|  | Breast | 10224 | 10224 |  | 4425 |  |  |  | 4425 | 43.28 |
|  | Liver | 3587 | 3587 |  |  | 1300 |  |  | 1300 | 36.24 |
|  | Upper GI | 9147 | 9147 |  |  |  | 1861 |  | 1861 | 20.35 |
|  | Colorectal | 11036 | 11036 |  |  |  |  | 1700 | 1700 | 15.40 |
|  | total | 48909 | 48909 | 5624 | 4425 | 1300 | 1861 | 1700 | 14910 | 30.49 |
| Group2 | Lung,Breast | 6088 | 12176 | 3481 | 3748 |  |  |  | 7229 | 59.37 |
|  | Lung,Liver | 2824 | 5648 | 1741 |  | 1430 |  |  | 3171 | 56.14 |
|  | Lung,Upper GI | 4591 | 9182 | 2185 |  |  | 1097 |  | 3282 | 35.74 |
|  | Lung,Colorectal | 5142 | 10284 | 2395 |  |  |  | 1098 | 3493 | 33.97 |
|  | Breast,Liver | 861 | 1722 |  | 509 | 440 |  |  | 949 | 55.11 |
|  | Breast,Upper GI | 2209 | 4418 |  | 1166 |  | 641 |  | 1807 | 40.90 |
|  | Breast,Colorectal | 1589 | 3178 |  | 883 |  |  | 492 | 1375 | 43.27 |
|  | Liver,Upper GI | 1301 | 2602 |  |  | 560 | 387 |  | 947 | 36.40 |
|  | Liver,Colorectal | 1064 | 2128 |  |  | 421 |  | 255 | 676 | 31.77 |
|  | Upper GI,Colorectal | 4761 | 9522 |  |  |  | 1352 | 1230 | 2582 | 27.12 |
|  | total | 30430 | 60860 | 9802 | 6306 | 2851 | 3477 | 3075 | 25511 | 41.92 |
| Group3 | Lung,Breast,Liver | 2158 | 6474 | 1567 | 1512 | 1303 |  |  | 4382 | 67.69 |
|  | Lung,Breast,Upper GI | 1738 | 5214 | 958 | 945 |  | 433 |  | 2336 | 44.80 |
|  | Lung,Breast,Colorectal | 1351 | 4053 | 780 | 818 |  |  | 384 | 1982 | 48.90 |
|  | Lung,Liver,Upper GI | 2024 | 6072 | 1300 |  | 1057 | 519 |  | 2876 | 47.36 |
|  | Lung,Liver,Colorectal | 1845 | 5535 | 1126 |  | 938 |  | 308 | 2372 | 42.85 |
|  | Lung,Upper GI,Colorectal | 5577 | 16731 | 2773 |  |  | 1487 | 1338 | 5598 | 33.46 |
|  | Breast,Liver,Upper GI | 454 | 1362 |  | 284 | 236 | 159 |  | 679 | 49.85 |
|  | Breast,Liver,Colorectal | 276 | 828 |  | 158 | 121 |  | 62 | 341 | 41.18 |
|  | Breast,Upper GI,Colorectal | 2254 | 6762 |  | 1202 |  | 532 | 461 | 2195 | 32.46 |
|  | Liver,Upper GI,Colorectal | 1467 | 4401 |  |  | 664 | 478 | 398 | 1540 | 34.99 |
|  | total | 19144 | 57432 | 8504 | 4919 | 4319 | 3608 | 2951 | 24301 | 42.31 |
| Group4 | Breast,Liver,Upper GI,Colorectal | 483 | 1932 |  | 297 | 261 | 167 | 132 | 857 | 44.36 |
|  | Lung,Liver,Upper GI,Colorectal | 7429 | 29716 | 4590 |  | 3900 | 1909 | 1572 | 11971 | 40.28 |
|  | Lung,Breast,Upper GI,Colorectal | 2188 | 8752 | 1278 | 1244 |  | 599 | 540 | 3661 | 41.83 |
|  | Lung,Breast,Liver,Colorectal | 1106 | 4424 | 805 | 791 | 682 |  | 164 | 2442 | 55.20 |
|  | Lung,Breast,Liver,Upper GI | 1363 | 5452 | 950 | 883 | 780 | 318 |  | 2931 | 53.76 |
|  | total | 12569 | 50276 | 7623 | 3215 | 5623 | 2993 | 2408 | 21862 | 43.48 |
| Group5 | Lung,Breast,Liver,Upper GI,Colorectal | 7787 | 38935 | 5056 | 4767 | 4426 | 1689 | 1302 | 17240 | 44.28 |
| total |  | 118839 | 256412 | 36609 | 23632 | 18519 | 13628 | 11436 | 103824 | 40.49 |


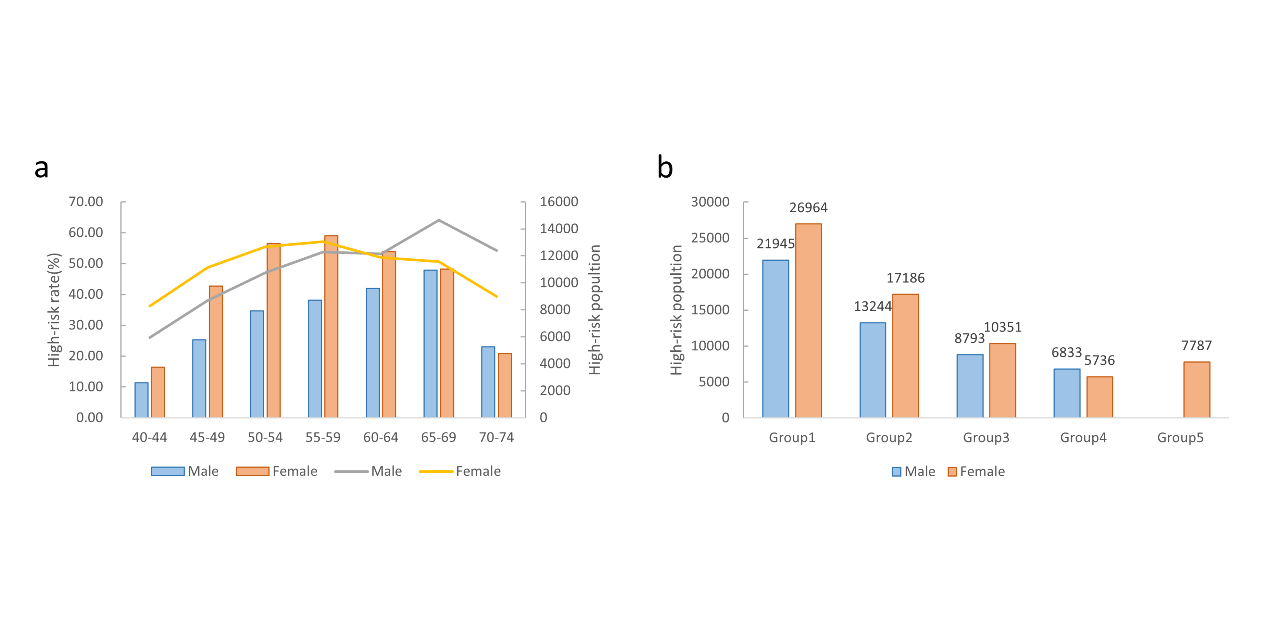


Supplementary Fig.S1. Distribution of gender, age, and the number of high-risk cancer types among high-risk population. a Number and high-risk rate of individuals in each age groups by gender. b Number of individuals in each high-risk group.
